# A sensitive colorimetric ELISA to measure allergen-specific salivary SIgA for monitoring sublingual immunity efficiency in pollinosis SLIT

**DOI:** 10.3389/falgy.2026.1857343

**Published:** 2026-10-05

**Authors:** Tetsuro Yamamoto, Fusako Mitsunaga, Atsushi Kotani, Kazuki Tajima, Muneki Hotomi, Shin Nakamura

**Affiliations:** 1 Research Center, EPS Holdings, Inc., Bunkyo-ku, Tokyo, Japan; 2 Research Center, EPS Innovative Medicine (Japan) Co., Ltd., Shinjuku-ku, Tokyo, Japan; 3 Intelligence and Technology Lab Inc., Kaizu, Gifu, Japan; 4 Biomedical Institute, NPO Primate Agora, Kaizu, Gifu, Japan; 5 Department of Otorhinolaryngology-Head and Neck Surgery, Wakayama Medical University, Wakayama, Japan

**Keywords:** allergen-specific SIgA, amplified colorimetric ELISA, pollinosis, saliva, sublingual immunotherapy

## Abstract

**Background:**

Sublingual immunotherapy (SLIT) is safe and one of the most effective etiological treatment for pollinosis. Allergen-specific salivary secretory immunoglobulin A (SIgA) is a useful marker to objectively evaluate treatment efficacy. However, saliva samples usually exhibit high background noise because of non-specific binding in enzyme-linked immunosorbent assays (ELISAs). We therefore aimed to establish an amplified ELISA to measure allergen-specific salivary SIgA levels to assess the efficacy of SLIT for Japanese cedar pollinosis.

**Methods:**

Biotinyl-tyramide (BT-Tyr) was used to amplify the signal in a colorimetric ELISA. The allergen of Japanese cedar pollen, Sugi basic protein (SBP), was biotinylated and used as a reporter to obtain sensitive and specific results with low background. To obtain preliminary observations, we applied this ELISA for measuring anti-SBP SIgA levels in saliva samples from SLIT-treated patients with Japanese cedar pollinosis.

**Results:**

The ELISA was highly sensitive and achieved more than 200-fold signal amplification compared with non-amplified conditions. It was also specific for antigens without background noise. The levels of SBP-specific SIgA in saliva increased in patients 9 months after SLIT initiation at the latest.

**Conclusions:**

We developed a sensitive colorimetric ELISA to measure SBP-specific SIgA in saliva using BT-Tyr-based amplification and biotinylated antigen as a reporter. We successfully applied this sensitive ELISA to monitor the levels of SBP-specific SIgA in saliva samples from patients with pollinosis receiving SLIT. Increased salivary SBP-specific SIgA levels were observed in these patients.

## 1 Introduction

Allergy causes various symptoms, including anaphylaxis, which is fatal in severe conditions (1). Even if not life-threatening, allergic symptoms reduce the quality of life of patients (2). As a curative treatment of allergy, allergen-specific immunotherapy (AIT) has been developed. The efficacy and safety of the treatment have been investigated via different routes. The major treatments are subcutaneous immunotherapy (SCIT), oral immunotherapy (OIT) and sublingual immunotherapy (SLIT) (3). Among these immunotherapies, SLIT has advantages over SCIT or OIT in terms of safety and self-administration, without the need for medical care personnel (4, 5), though the treatment period of SLIT is usually long and the curing process is slow (6, 7).

To evaluate the efficacy and investigate the mechanism of SLIT, monitoring the levels of anti-allergen antibodies during the time course of treatment is required. For example, decreasing the levels of allergen-specific IgE and increasing the levels of allergen-specific IgG4 are related to the amelioration of the symptoms (8, 9). Among antibodies, secretory immunoglobulin A (SIgA) exhibits a unique function associated with its structure. SIgA is polymeric, mainly dimers, and is complexed with the secretory component (10, 11). Due to the secretory component masking the susceptible and vulnerable part of enzymatic digestion of IgA, SIgA is remarkably stable in environments where other antibodies would be degraded (12). SIgA plays an important role in immune tolerance against commensal bacteria and possible antigens in food (13, 14). In previous studies, relationship between SIgA levels in saliva and SLIT has been suggested (14, 15). Anti-allergen SIgA could therefore be a potential biomarker for monitoring AIT efficacy.

Saliva contains a large number of microorganisms, including bacteria (13), which are not in circulating blood physiologically. In saliva, there are abundant lytic enzymes including matrix metalloproteases or lysozymes (16). These components in saliva could influence the degradation or digestion of immunoglobulins or immunoglobulin-like molecules and also materials used in the assay, resulting in conformational changes of molecules or even producing neo-antigens. Thus, saliva contains many obstacles to detect analyte by enzyme-linked immunosorbent assays (ELISA). To reduce non-specific binding caused by these obstacles, a sensitive and specific method is required.

In previous studies detecting antigen-specific IgA (SIgA), saliva samples were assayed at low dilutions, usually up to 1:160. Similarly, in studies of upper respiratory infectious diseases, saliva samples were measured at dilutions of 1:2 to 1:160 to detect anti-SARS-CoV-2 SIgA (17–21). Lower dilutions of saliva samples produce higher background noise because of non-specific binding. In these ELISA methods, antigens were immobilized on the plates and antigen-specific SIgA in saliva was detected by labeled detector antibodies. Tsukinoki et al. improved the sensitivity of ELISA to detect anti-SARS-CoV-2 SIgA in saliva by using a biotin-labeled antigen for detection. The dilution of saliva was 1:500 in these studies. As in the method by Tsukinoki et al., the sensitivity was increased when ELISA wells were coated with capture antibodies and specific SIgAs in saliva was detected by biotinylated antigens (22, 23).

Concerning sensitivity, Bobrow et al. reported a drastic signal amplification method applicable to ELISA that utilizes biotinyl-tyramide (BT-Tyr) (24). Importantly, the extent of signal amplification by BT-Tyr correlates with the amount of immobilized analyte before amplification, so that quantitativeness is guaranteed.

In this study, we examined this amplification method in detail to find the optimal concentrations of hydrogen peroxide (H_2_O_2_) and BT-Tyr to measure allergen-specific IgA. Furthermore, we biotinylated allergen and used it as a reporter to reduce non-specific binding of saliva samples.

As a pilot study, here we applied this amplified ELISA to measure allergen-specific SIgA in saliva from patients with pollinosis caused by Japanese cedar pollen (25) who were receiving SLIT. The patients with pollinosis suffer from nasal and/or ocular symptoms and reduced quality of life (26). The allergen of this pollen is Sugi basic protein (SBP), which is a complex of the major antigens (Cry j1 and Cry j2) (27). This allergen, SBP was biotinylated and used in this study.

## 2 Materials and methods

Figure 1 shows an outline of the ELISA materials, procedures, and conditions.

**Figure 1:**
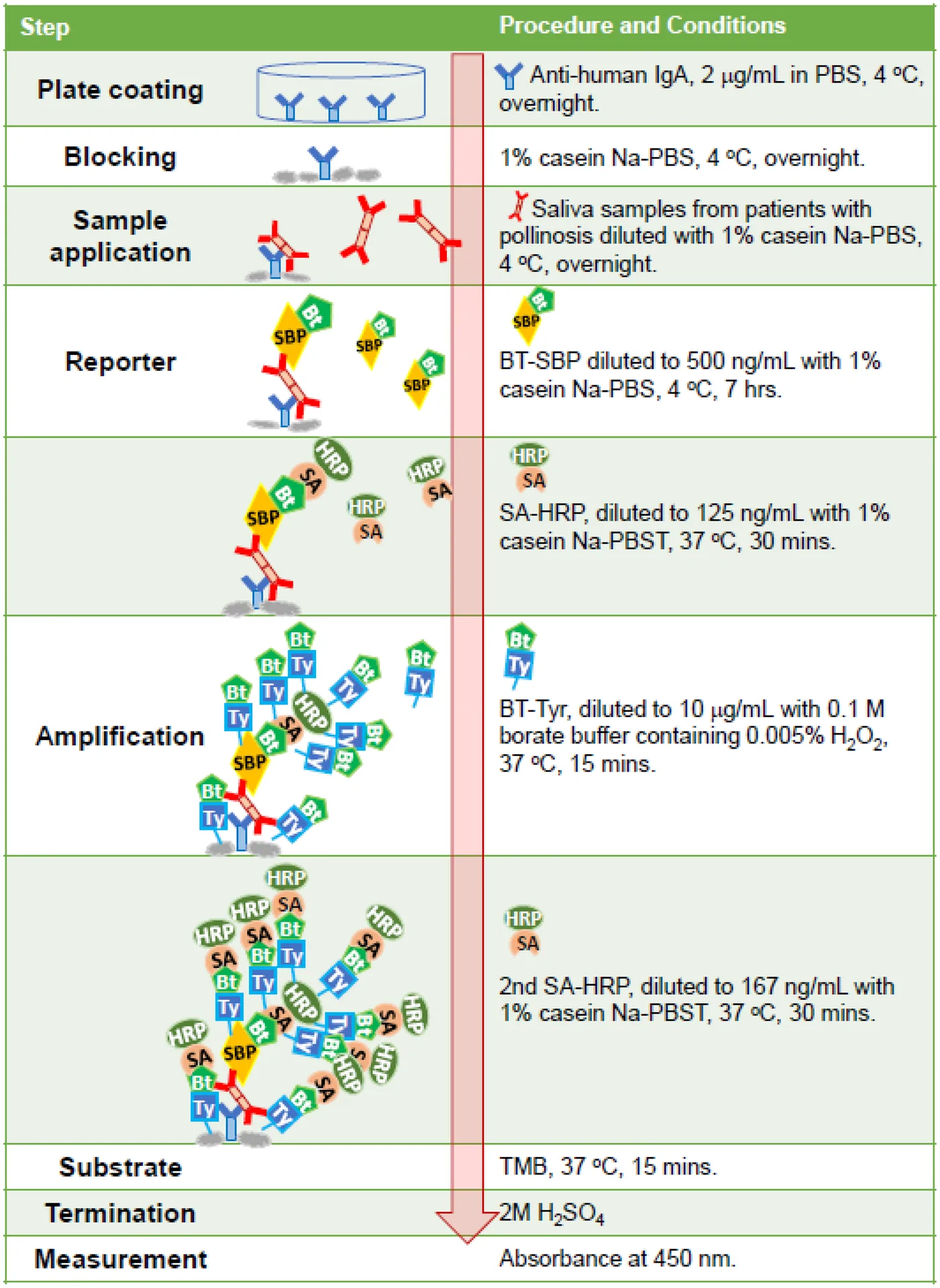
Outline of the amplified ELISA to measure antigen-specific SIgA in saliva samples utilizing biotinylated antigen (BT-SBP). ELISA, enzyme-linked immunosorbent assay; SIgA, secretory immunoglobulin A; SBP, Sugi basic protein; BT-SBP, biotinylated SBP; SA-HRP, streptavidin-HRP; BT-Tyr, biotinyl-tyramide; TMB, tetramethylbenzidine.

### 2.1 ELISA materials

The primary materials used for the amplified ELISA were as follows. SBP (BioDynamics Laboratory Inc., Tokyo, Japan) was biotinylated using a Biotin Labeling Kit-NH_2_ (Dojindo Laboratories, Kumamoto, Japan). Because the molecular weights of the major antigens, Cry j1 and Cry j2 in SBP are less than 50 kDa (Cry j1: 50, 30, 17 kDa; Cry j2: 38 kDa) (27), while filter size of the kit filtration tube is >50 kDa, a centrifugal tube with a molecular weight cut-off of 10 kDa (Amicon Ultra, Merck KGaA, Darmstadt, Germany) was used instead of the kit filtration tube. The concentration of biotinylated SBP (BT-SBP) was determined using a DC Protein Assay (Bio-Rad, Hercules, CA, USA).

BT-Tyr (Sigma-Aldrich, Burlington, MA, USA) was dissolved in dimethyl sulfoxide as a stock solution (50 mg/mL) and divided into several small volumes for one-time use, followed by freezing. The working solution of BT-Tyr was prepared by diluting the stock solution to a final concentration of 1 mg/mL with ethanol, after which it was kept at 4 °C until use. Prior to use of the diluted BT-Tyr, a 30% H_2_O_2_ solution (Fujifilm Wako Pure Chemical Corporation, Osaka, Japan) was diluted to a final concentration of 0.005% with 0.1 M borate buffer (pH 8.5). The diluted BT-Tyr was then added to borate buffer containing H_2_O_2_ to prepare a final concentration of 10 μg/mL, which was used immediately. A BT-Tyr concentration of 10 μg/mL yielded the best amplification, with high sensitivity and low background noise.

### 2.2 ELISA procedures

The outline of the procedure and conditions for this amplified ELISA are shown in Figure 1. The principle of the method introduced by Bobrow et al. (24) is as follows. The plate is blocked with proteins that contain electron-rich moieties such as tyrosine or tryptophan residues. Horseradish peroxidase (HRP) is used as both a reporter and a catalytic enzyme, in the form of HRP-labeled antibodies or streptavidin conjugated with HRP (streptavidin-HRP). After this reporter-material conjugated with HRP is immobilized on the plate, a biotin-labeled phenols such as BT-Tyr is added along with H_2_O_2_. The HRP then catalyzes biotinylated phenolic compounds to deposit on the surface of the wells by covalent binding via free-radical formation and subsequent reaction with the electron-rich moieties of the blocking protein. Next, newly added streptavidin-HRP binds immobilized biotin of BT-Tyr, resulted in clump of reporter deposition.

#### 2.2.1 Plate coating

Polystyrene microplates (Maxisorp, Nunc, Roskilde, Denmark) were coated with 100 µL/well of anti-human IgA (polyclonal, goat; Mabtech, Stockholm, Sweden) diluted to a final concentration of 2 μg/mL in phosphate-buffered saline (PBS) containing 0.02% sodium azide, pH 7.4. The plates were then incubated overnight at 4 °C. The plates were washed with 0.05% Tween 20-PBS (PBST) at each step, except for the final step, which involved the addition of the stop solution to the substrate.

#### 2.2.2 Blocking

After incubation, the plates were washed twice with 300 µL/well of 0.05% PBST, and blocked with 200 µL/well of 1% casein sodium (casein-Na; Fujifilm Wako Pure Chemical Corporation), overnight at 4 °C. It is important that the blocking proteins contain electron-rich moieties such as tyrosine or tryptophan residues, for the “Amplification” step described below (24). Blocking with casein resulted in a lower background signal than blocking with bovine serum albumin (BSA). BSA typically caused high absorbance values (>0.1) in wells containing only the blocking agent was immobilized, whereas casein showed lower absorbance in amplification ELISA. For example, in our preliminary amplification test, the absorbance of blank wells blocked with casein and BSA were 0.041 ± 0.001 and 0.143 ± 0.053, respectively (mean ± SEM, *n* = 16). These results were in accordance with previous studies which showed superiority of casein as a blocking agent compared with BSA (28, 29). This is mostly because of the heterogeneous molecular size of proteins in casein (30), especially with small sizes, which can effectively cover the wells without disturbing immobilized capture antigens or antibodies.

#### 2.2.3 Sample application

Saliva samples were diluted with 1% casein in Na-PBS. Blocked plates were washed twice with 300 µL/well of PBST, and the diluted samples (100 µL/well) were applied to the plates. The plates were incubated overnight at 4 °C.

#### 2.2.4 Reporter

After incubation, the plates were washed four times with 300 µL/well of PBST. BT-SBP diluted to a final concentration of 500 ng/mL in 1% casein-Na-PBS was added and bound to the immobilized anti-SBP SIgA. The plates were incubated for 7 h at 4 °C.

Next, the plates were washed four times with 300 µL/well of PBST, and streptavidin-conjugated horseradish peroxidase (SA-HRP; Invitrogen-Thermo Fisher Scientific Inc., Waltham, MA, USA) diluted to a final concentration of 125 ng/mL with 1% casein-Na-PBST was added. The plates were subsequently incubated for 30 min at 37 °C.

#### 2.2.5 Amplification

After the first SA-HRP incubation, the plates were washed four times with 300 µL/well of PBST. BT-Tyr, diluted to a final concentration of 10 μg/mL in 0.1 M borate buffer, pH 8.5, containing 0.005% H_2_O_2_, was added, and the plates were incubated for 15 min at 37 °C. During this step, the HRP of the already immobilized SA-HRP catalyzes the BT-Tyr to deposit onto the surface of the wells by covalent binding via free-radical formation and reaction with electron-rich moieties of the blocking protein (24).

The plates were then washed four times with 300 µL/well of PBST, and second SA-HRP diluted to a final concentration of 167 ng/mL was added. During this step, the newly added streptavidin-HRP binds immobilized biotin of BT-Tyr, resulted in clump of reporter deposition. Thus, the signal is amplified, correlating with the amount of immobilized analyte before amplification (24). The plates were then incubated for 30 min at 37 °C.

#### 2.2.6 Substrate and terminating reaction

Finally, the plates were washed five times with 300 µL/well of PBST, and the chromogenic substrate, 3,3′,5,5′-tetramethylbenzidine (TMB; Sigma-Aldrich), was added to develop the color at 37 °C usually for 15 min. The reaction was then terminated by adding 2M H_2_SO_4_.

#### 2.2.7 Colorimetric measurement

The absorbance at 450 nm (with reference at 630 nm) was measured using a plate reader in the visible light range (iMark Microplate Absorbance Reader, Bio-Rad).

### 2.3 SBP blocking (absorption) test for specificity

Saliva samples typically produce greater background noise than serum or plasma samples because of non-specific binding. To confirm the specificity of the amplified ELISA using BT-SBP as a reporter for measuring anti-SBP SIgA in saliva, an SBP blocking (absorption) test was performed.

Wells of an ELISA plate were coated with either anti-human IgA as described above in Section 2.2.1 , or with SBP at 2 μg/mL for comparison. Next, the wells were blocked as described above in Section 2.2.2.

For the preincubation with saliva samples, SBP was diluted to various concentrations (0–4 μg/mL) in 1% casein-Na-PBS. Saliva samples obtained from patients with pollinosis treated with SLIT were pooled and diluted 1:400 in 1% casein-Na-PBS. The diluted SBP samples were mixed with the saliva samples and preincubated for 90 min at room temperature.

The preincubated saliva samples were then added to either capture the antibody (anti-human IgA)- or antigen (SBP)-coated wells. For antibody-coated wells, anti-SBP SIgA in the saliva samples was detected using BT-SBP as described in Section 2, whereas for antigen-coated wells, anti-SBP SIgA in the saliva samples was detected using biotinylated anti-IgA (polyclonal, goat; Mabtech). The subsequent amplification procedures were the same as those described in Section 2. The blocking effect of a certain concentration (Conc) of preincubated SBP was calculated from the resulting absorbance (Abs) as follows: Blockingeffect%=(1−(AbsofConcX/AbsofConc0))×100.

### 2.4 Study population

We applied this amplified ELISA to monitor salivary anti-SBP SIgA levels in saliva samples from Japanese patients with Japanese cedar pollinosis treated with SLIT using CEDARCURE (Japanese Cedar Pollen Sublingual Tablets, Torii Pharmaceutical Co. Ltd., Tokyo, Japan) (6). Twenty-two patients treated with SLIT and five patients without SLIT were enrolled. Saliva samples were collected at otolaryngology hospitals from patients at 3-month intervals for 12 months. In the SLIT group, patients were administered 2,000 JAU of CEDARCURE once daily for the first week after the beginning of treatment. The dose was then increased to 5,000 JAU/d from the second week onward. The patients were carefully examined and observed by physicians during the first administration and at periodic follow-up visits. Among the patients enrolled, six patients with SLIT and two patients without SLIT were omitted from ELISA analyses because of an insufficient number of visits to the hospital for saliva collection. Informed consent and sample collection were conducted under the arrangement of Wakayama Medical University. All sampling procedures were approved by the Research Ethics Committee of Wakayama Medical University (approval number: 3218; date: 31 July 2021).

### 2.5 Statistical analysis

The Mann–Whitney *U*-test was used to compare the levels of saliva SBP-specific SIgA between patients treated with SLIT and without SLIT at certain time points. Z-scores were calculated using means of U values (ranking) and variance of U values. Then *p*-values were calculated using function of normal distribution of Z-scores. A *p*-value of <0.05 was considered significant.

## 3 Results and discussion

### 3.1 Specificity and sensitivity of the amplified ELISA

#### 3.1.1 Specificity

We confirmed the specificity of the amplified ELISA using an adsorption test in which pooled saliva samples diluted 1:400 were preincubated with various concentrations of SBP prior to application to the coated plates.

As shown in Figure 2, preincubating SBP at a concentration of at least 0.8 μg/mL was sufficient to absorb anti-SBP SIgA in the saliva samples when BT-SBP was used as a reporter in the antibody-coated wells (indicated by red circles). However, when anti-IgA-BT was used as the reporter in the SBP-coated wells, the blocking effect of preincubation with SBP was <80% even at 4 μg/mL (black triangles) because of the non-specific binding effect.

**Figure 2:**
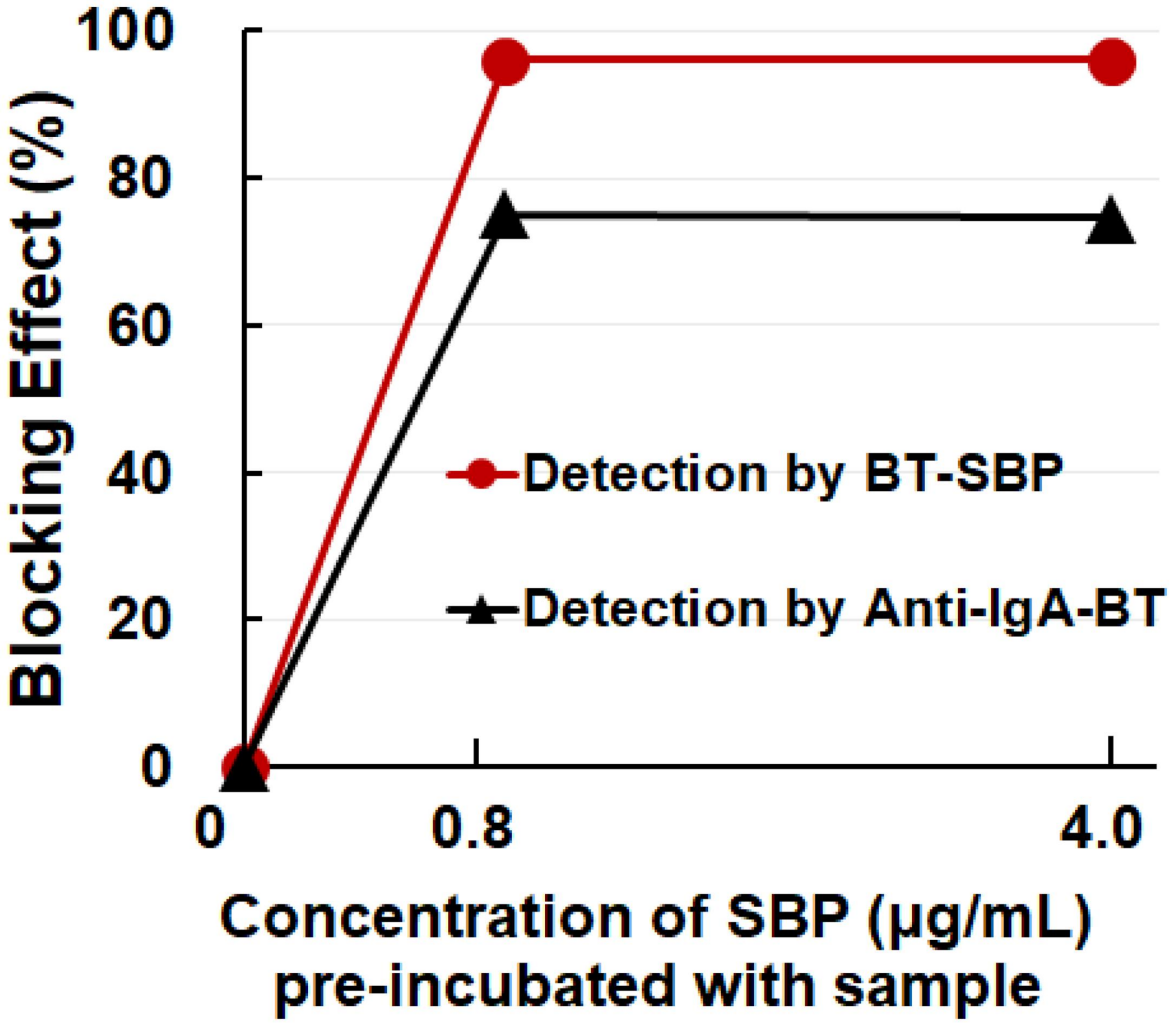
Specificity of the amplified ELISA for measuring antigen-specific SIgA in saliva samples using biotinylated antigen (BT-SBP). Saliva samples from a patient with pollinosis treated with SLIT were preincubated with SBP diluted at concentrations of 0 to 4 μg/mL. Anti-SBP SIgA in the preincubated samples was measured by the amplified ELISA using either BT-SBP (red circles) or biotinylated anti-IgA (black triangles). The blocking effect of a certain concentration (Conc) of preincubated SBP was calculated from the resulting absorbance (Abs) as follows: Blockingeffect%=(1−(AbsofConcX/AbsofConc0))×100. ELISA, enzyme-linked immunosorbent assay; SIgA, secretory immunoglobulin A; SBP, Sugi basic protein; BT-SBP, biotinylated SBP; SLIT, sublingual immunotherapy; Anti-IgA-BT, biotinylated anti-IgA.

#### 3.1.2 Sensitivity

The amplified ELISA was compared with the non-amplified method using serially diluted saliva samples from three patients treated with SLIT.

The amplified method was highly sensitive and achieved more than 200-fold signal amplification compared with the non-amplified condition (Figure 3). The raw values of blank absorbance were low in both cases (0.064 ± 0.013 and 0.031 ± 0.003, amplified and non-amplified, respectively) owing to casein-blocking. When the Limit of Detection (LOD, blank mean + 3SD) values were calculated, they were 0.104 and 0.039 for amplified and non-amplified ELISA, respectively. Before subtraction of the blank Optical Density (OD), the raw data for the 12,000-fold diluted sample were above the LOD and could be distinguished from the blank value. When saliva samples diluted less than 200 times, the reading maxed out in the amplified ELISA. In Figure 3, the sample data were obtained by subtraction of blank value and drawn as the mean value of three tests.

**Figure 3:**
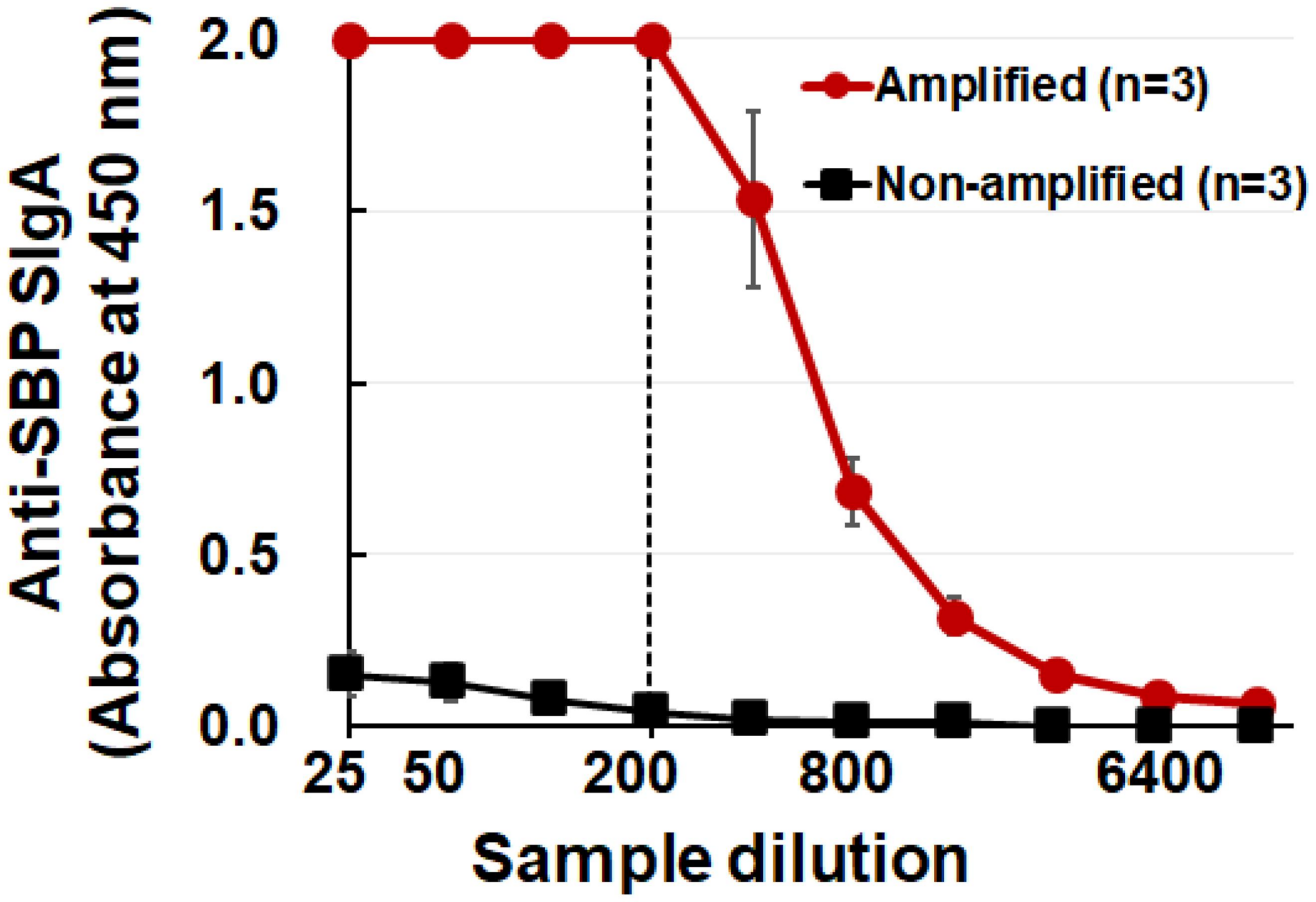
Sensitivity of the amplified ELISA to measure antigen-specific SIgA in saliva samples utilizing biotinylated antigen (BT-SBP). Saliva samples from three patients with pollinosis treated with SLIT were serially diluted to a range of 1:25 to 1:12,800. The anti-SBP SIgA in the patient's diluted samples was measured using the BT-Tyr amplification ELISA (red circles) and compared with the non-amplified ELISA (black squares). The data are expressed as mean value and SEM. ELISA, enzyme-linked immunosorbent assay; SIgA, secretory immunoglobulin A; SLIT, sublingual immunotherapy; SBP, Sugi basic protein; BT-SBP, biotinylated SBP; BT-Tyr, biotinyl-tyramide.

### 3.2 Application

We applied this amplified ELISA to all samples from patients who provided saliva for 12 months. Each saliva sample was diluted at 1:450 with 1% casein-Na-PBS. Among the patients with SLIT, there were individual differences in the pattern of anti-SBP SIgA levels. Patients treated without SLIT showed no increase in specific SIgA. No samples from these non-SLIT patients exceeded 0.2 of absorbance throughout the study period. Thus, the absorbance of 0.2 was set as a temporary cut-off in this study. When we define patients who showed an increase in specific SIgA over 0.2 of absorbance at any time point as “responders” in terms of mucosal immune response, there were 11 responders (69% of analyzed SLIT patients) (Figure 4, red circles). The other five patients with SLIT (31% of analyzed SLIT patients) did not show an increase in specific SIgA over 0.2 of absorbance (Figure 4, green triangles). We could define the patients as “non-responders” in contrast to the “responders” here. These individual differences in the pattern of specific SIgA can be useful information to monitor the efficacy of SLIT treatment and consider the differences in immune response of patients individually. However, there was no increase in SBP-specific SIgA levels in patients with pollinosis who did not receive SLIT as described above (Figure 4, black diamonds).

**Figure 4:**
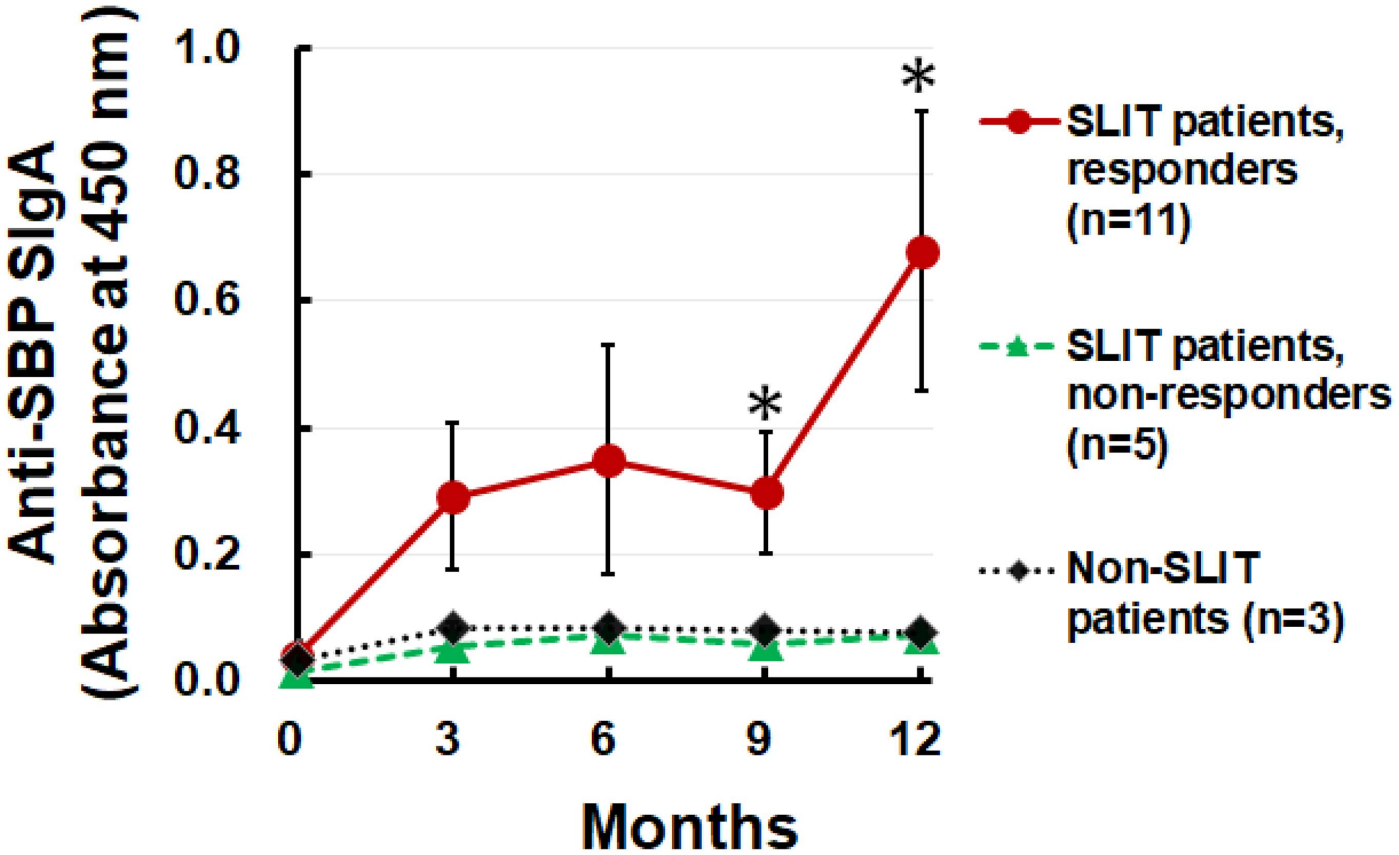
Saliva SBP-specific SIgA in patients with pollinosis followed up over a 12-month period of SLIT. Each saliva sample was diluted at 1:450 with 1% casein-Na-PBS. There were individual differences in the pattern of specific SIgA in patients treated with SLIT. Eleven patients with SLIT showed an increase in specific SIgA of at least 0.2 in absorbance at least one time point (red circles). The other five patients with SLIT did not show an increase of more than 0.2 in absorbance throughout the study period (green triangles). Patients without SLIT did not show any increase in specific SIgA (black diamonds). The data are expressed as mean value and SEM. *Statistically significant between SLIT responders vs. SLIT non-responders and SLIT responders vs. non-SLIT patients by Mann–Whitney *U*-test. At 9-month point, *p* = 0.002 and *p* = 0.036, SLIT responders vs. SLIT non-responders and SLIT responders vs. non-SLIT patients respectively. At 12-month point, *p* = 0.0004 and *p* = 0.0014, SLIT responders vs. SLIT non-responders and SLIT responders vs. non-SLIT patients respectively. SBP, Sugi basic protein; SIgA, secretory immunoglobulin A; SLIT, sublingual immunotherapy.

The levels of anti-SBP SIgA in SLIT responders at 9 and 12 months after the beginning of treatment were significantly higher than those in SLIT non-responders or Non-SLIT patients (at 9-month point, *p* = 0.002 and *p* = 0.036, SLIT responders vs. SLIT non-responders and SLIT responders vs. non-SLIT patients, respectively) (at 12-month point, *p* = 0.0004 and *p* = 0.0014, SLIT responders vs. SLIT non-responders and SLIT responders vs. non-SLIT patients, respectively).

### 3.3 Limitation and further application

A limitation of our study is the small sample size. To evaluate the effectiveness of the amplified ELISA, the number of patients was insufficient. It is necessary to increase the number of patients for statistical analyses. With a statistically sufficient sample size, this amplified ELISA could be one of the useful tools to evaluate the efficacy of SLIT. For wider application, it is required to configure an arbitrary unit with a standard curve generated by serially diluting a high-titer saliva sample to minimize inter-assay variation.

This amplification method could be applied to measure not only SBP-specific antibodies but also other allergen-specific antibodies. Most allergens are proteins that can be biotinylated using a commercially available labeling kit. Using these biotinylated allergens and BT-Tyr, it is possible to develop a sensitive ELISA to measure a certain allergen-specific SIgA in saliva.

The advantages of the amplified ELISA include not only its high sensitivity, but also its versatility. The necessary reagents can be purchased commercially, and the absorbance is measurable with a conventional chromogenic plate reader, which offers greater availability and affordability compared with fluorescence or chemiluminescence readers.

It could be beneficial for patients and doctors to obtain the antibody information with other biomarkers or immunological factors to discuss the treatment plan in the context of SLIT.

## 4 Conclusion

We established a sensitive colorimetric ELISA to measure salivary SBP-specific SIgA levels in saliva. The most critical point is the amplification by BT-Tyr after the first SA-HRP. Furthermore, biotinylated SBP was used to detect specific SIgA in the wells where capture antibodies were immobilized. Sodium casein was used as an effective blocking agent with low background, and electron-rich amino acids such as tyrosine or tryptophan in casein provide covalent binding during amplification by BT-Tyr. These procedures enable the obtainment of sensitive and specific results to measure specific SIgA in saliva. This method could be applicable to detect SIgA specific to other allergens or antigens provided that the target allergens or antigens are properly biotinylated. The resulting absorbance can be measured using a chromogenic plate reader in the visible light range. We applied this sensitive ELISA to monitor levels of SBP-specific SIgA in saliva samples from patients with pollinosis who received SLIT for 12 months. Using this ELISA, an increase in salivary SBP-specific SIgA levels was observed within 9 months after SLIT initiation at the latest. However, a limitation of this study is the small sample size. It is necessary to increase the number of patients for statistical analyses. In the future, the correlation between levels of allergen-specific SIgA in saliva and treatment efficacy (such as amelioration of symptoms) is required to be investigated. In addition, this ELISA could be useful to monitor patients who do not respond to the treatment.

## Data Availability

The raw data supporting the conclusions of this article will be made available by the authors, without undue reservation.

## References

[1] Dribin TE Motosue MS Campbell RL. Overview of allergy and anaphylaxis. Emerg Med Clin North Am. (2022) 40(1):1–17. 10.1016/j.emc.2021.08.007 34782082 PMC8604419

[2] Small M Piercy J Demoly P Marsden H. Burden of illness and quality of life in patients being treated for seasonal allergic rhinitis: a cohort survey. Clin Transl Allergy. (2013) 3(1):33. 10.1186/2045-7022-3-33 24107462 PMC3852977

[3] Schoos A-MM Bullens D Chawes BL Costa J De Vlieger L DunnGalvin A. Immunological outcomes of allergen-specific immunotherapy in food allergy. Front Immunol. (2020) 11:568598. 10.3389/fimmu.2020.568598 33224138 PMC7670865

[4] Nomura Y Okubo K Nakamura T Sawaki S Kitagou H Idei N. Long-term treatment of Japanese cedar pollinosis with Japanese cedar pollen SLIT drops and persistence of treatment effect: a post-marketing clinical trial. Allergol Int. (2021) 70(1):96–104. 10.1016/j.alit.2020.05.008 32653209

[5] Gotoh M Kaminuma O Okubo K. From the genesis to the present: the evolution of sublingual immunotherapy for cedar pollinosis. Allergol Int. (2024) 73(4):494–500. 10.1016/j.alit.2024.07.001 39043490

[6] Huoman J Papapavlou G Pap A Alm J Nilsson LJ Jenmalm MC. Sublingual immunotherapy alters salivary IgA and systemic immune mediators in timothy allergic children. Pediatr Allergy Immunol. (2019) 30(5):522–30. 10.1111/pai.13047 30803044

[7] Fujii T Kitamura Y Kamimura S Takeda N. Effects of sublingual immunotherapy with tablets or drops containing Japanese cedar pollen antigens on nasal symptoms and sleep disturbance in patients with Japanese cedar pollinosis. J Med Invest. (2022) 69(1.2):97–100. 10.2152/jmi.69.97 35466153

[8] Sola Martínez FJ Barranco Jiménez RM Martín García C Senent Sánchez C Blanco Guerra C Fernández-Rivas M. Intradermal phleum pratense allergoid immunotherapy. Double-blind, randomized, placebo-controlled trial. Clin Exp Allergy. (2020) 50(12):1352–61. 10.1111/cea.13740 PMC7756767 32946612

[9] Ojeda P Barjau MC Subiza J Moreno A Ojeda I Solano E. Grass pollen allergoids conjugated with mannan for subcutaneous and sublingual immunotherapy: a dose-finding study. Front Immunol. (2024) 15:1431351. 10.3389/fimmu.2024.1431351 38989287 PMC11233432

[10] Corthésy B. Multi-faceted functions of secretory IgA at mucosal surfaces. Front Immunol. (2013) 4:185. 10.3389/fimmu.2013.00185 PMC3709412 23874333

[11] Brandtzaeg P. Secretory IgA: designed for anti-microbial defense. Front Immunol. (2013) 4:222. 10.3389/fimmu.2013.00222 23964273 PMC3734371

[12] Bonner A Almogren A Furtado PB Kerr MA Perkins SJ. The nonplanar secretory IgA2 and near planar secretory IgA1 solution structures rationalize their different mucosal immune responses. J Biol Chem. (2009) 284(8):5077–87. 10.1074/jbc.M807529200 19109255 PMC2643523

[13] Lynge Pedersen AM Belstrøm D. The role of natural salivary defences in maintaining a healthy oral microbiota. J Dent. (2019) 80(Suppl 1):S3–12. 10.1016/j.jdent.2018.08.010 30696553

[14] Kulis M Saba K Kim EH Bird JA Kamilaris N Vickery BP. Increased peanut-specific IgA levels in saliva correlate with food challenge outcomes after peanut sublingual immunotherapy. J Allergy Clin Immunol. (2012) 129(4):1159–62. 10.1016/j.jaci.2011.11.045 22236732 PMC3763925

[15] Haruna T Kariya S Fujiwara T Yuta A Higaki T Zhao P. Role of whole saliva in the efficacy of sublingual immunotherapy in seasonal allergic rhinitis. Allergol Int. (2019) 68(1):82–9. 10.1016/j.alit.2018.07.008 30166059

[16] Maciejczyk M Pietrzykowska A Zalewska A Knaś M Daniszewska I. The significance of matrix metalloproteinases in oral diseases. Adv Clin Exp Med. (2016) 25(2):383–90. 10.17219/acem/30428 27627574

[17] Isho B Abe KT Zuo M Jamal AJ Rathod B Wang JH. Persistence of serum and saliva antibody responses to SARS-CoV-2 spike antigens in COVID-19 patients. Sci Immunol. (2020) 5(52):eabe5511. 10.1126/sciimmunol.abe5511 33033173 PMC8050884

[18] Costantini VP Nguyen K Lyski Z Novosad S Bardossy AC Lyons AK. Development and validation of an enzyme immunoassay for detection and quantification of SARS-CoV-2 salivary IgA and IgG. J Immunol. (2022) 208(6):1500–8. 10.4049/jimmunol.2100934 35228262 PMC8916996

[19] Thomas AC Oliver E Baum HE Gupta K Shelley KL Long AE. Evaluation and deployment of isotype-specific salivary antibody assays for detecting previous SARS-CoV-2 infection in children and adults. Commun Med (Lond). (2023) 3(1):37. 10.1038/s43856-023-00264-2 36922542 PMC10016188

[20] Akeel S Almazrooa S Jazzar A Mohammed Sindi A Farsi NJ Binmadi N. Detection of specific immunoglobulins in the saliva of patients with mild COVID-19. Cureus. (2024) 16(1):e52113. 10.7759/cureus.52113 38213933 PMC10783611

[21] Bhavsar D Sano K Singh G Krammer F. An ELISA-based method to measure mucosal antibody responses against SARS-CoV-2 in human saliva. Curr Protoc. (2024) 4(4):e1024. 10.1002/cpz1.1024 38578049

[22] Tsukinoki K Yamamoto T Saito J Sakaguchi W Iguchi K Inoue Y. Prevalence of saliva immunoglobulin A antibodies reactive with severe acute respiratory syndrome coronavirus 2 among Japanese people unexposed to the virus. Microbiol Immunol. (2022) 66(8):403–10. 10.1111/1348-0421.13011 35607844 PMC9347685

[23] Tsukinoki K Yamamoto T Handa K Iwamiya M Saruta J Ino S. Detection of cross-reactive immunoglobulin A against the severe acute respiratory syndrome-coronavirus-2 spike 1 subunit in saliva. PLoS One. (2021) 16(11):e0249979. 10.1371/journal.pone.0249979 34813596 PMC8610234

[24] Bobrow MN Harris TD Shaughnessy KJ Litt GJ. Catalyzed reporter deposition, a novel method of signal amplification. Application to immunoassays. J Immunol Methods. (1989) 125(1-2):279–85. 10.1016/0022-1759(89)90104-x 2558138

[25] Fujimura T Kawamoto S. Spectrum of allergens for Japanese cedar pollinosis and impact of component-resolved diagnosis on allergen-specific immunotherapy. Allergol Int. (2015) 64(4):312–20. 10.1016/j.alit.2015.05.008 26433527

[26] Hashiguchi K Tang H Fujita T Tsubaki S Fujita M Suematsu K. Preliminary study on Japanese cedar pollinosis in an artificial exposure chamber (OHIO chamber). Allergol Int. (2007) 56(2):125–30. 10.2332/allergolint.O-06-453 17384532

[27] Wang Q Morita J Gong X Nakamura S Suzuki M Lu S. Characterization of the physical form of allergenic cry j 1 in the urban atmosphere and determination of Cryj 1 denaturation by air pollutants. Asian J Atmos Environ. (2012) 6:33–40. 10.5572/ajae.2012.6.1.033

[28] Jiang X Wu M Albo J Rao Q. Non-specific binding and cross-reaction of ELISA: a case study of porcine hemoglobin detection. Foods. (2021) 10(8):1708. 10.3390/foods10081708 34441486 PMC8394222

[29] Andriola DD Ferreira GN Candelário JM Piazenski IN Biavatti M Sakamoto LB. Validated cost-effective casein-based blocking buffers for enhanced neurocysticercosis ELISA accuracy. Diagn Microbiol Infect Dis. (2025) 113(4):117033. 10.1016/j.diagmicrobio.2025.117033 40752290

[30] Bhat MY Dar TA Singh LR. Casein proteins: structural and functional aspects. In: Gigli I. (Ed.). Milk Proteins—from Structure to Biological Properties and Health Aspects. London: InTech (2016). 10.5772/64187

